# Protective Effect of Anthocyanin on Neurovascular Unit in Cerebral Ischemia/Reperfusion Injury in Rats

**DOI:** 10.3389/fnins.2018.00947

**Published:** 2018-12-11

**Authors:** Zihao Pan, Mengdi Cui, Guoliang Dai, Tianjie Yuan, Yuhua Li, Tuo Ji, Yang Pan

**Affiliations:** ^1^College of Pharmacy, Nanjing University of Chinese Medicine, Nanjing, China; ^2^Thyroid and Breast Surgery, The Third Affiliated Hospital of Nanjing University of Chinese Medicine, Nanjing, China; ^3^Department of Clinical Pharmacology, The Affiliated Hospital of Nanjing University of Chinese Medicine, Nanjing, China

**Keywords:** cerebral ischemia/reperfusion injury, neurovascular unit, anthocyanin, apoptosis, inflammation

## Abstract

Treating cerebral ischemia continues to be a clinical challenge. Studies have shown that the neurovascular unit (NVU), as the central structural basis, plays a key role in cerebral ischemia. Here, we report that anthocyanin, a safe and natural antioxidant, could inhibit apoptosis and inflammation to protect NVU in rats impaired by middle cerebral artery occlusion/reperfusion (MCAO/R). Administration of anthocyanin significantly reduced infarct volume and neurological scores in MCAO/R rats. Anthocyanin could also markedly ameliorate cerebral edema and reduce the concentration of Evans blue (EB) by inhibiting MMP-9. Moreover, anthocyanin alleviated apoptotic injury resulting from MCAO/R through the regulation of Bcl-2 family proteins. The levels of inflammation-related molecules including tumor necrosis factor-α (TNF-α), interleukin-1β (IL-1β), and interleukin-6 (IL-6), which were over-expressed with MCAO/R, were decreased by anthocyanin. In addition, Nuclear factor-kappa B (NF-κB) and the NLRP3 inflammasome pathway might be involved in the anti-inflammatory effect of anthocyanin. In conclusion, anthocyanin could protect the NVU through multiple pathways, and play a protective role in cerebral ischemia/reperfusion injury.

## Introduction

Treating cerebral ischemia continues to be a clinical challenge and underlying mechanisms of cerebral ischemia remain elusive. Recent insight into the basic mechanism involved in cerebral ischemia indicates that the neurovascular unit (NVU) is a new target for the overall study of neuronal damage and protection mechanisms (Lake et al., 2017), which consists of neurons and blood-brain barrier (BBB), including astrocytes, microglia, vascular endothelial cells and extracellular matrix (ECM; Maki et al., 2013). The destruction of the BBB promotes the development of neurological dysfunction in cerebral ischemia (Rosenberg, 2012). MMP-2 and MMP-9, which are the most important ECM degrading enzymes *in vivo*, are most closely related to the destruction of the BBB (Turner and Sharp, 2016). The use of MMP-9 inhibitors can significantly reduce cerebral edema and cerebral infarct volume caused by ischemia, and reduce BBB damage (Turner and Sharp, 2016).

Apoptosis and the inflammatory response are important processes that cause NVU destruction, and they play a key role in the pathology of stroke (Vidale et al., 2017). In stroke cases, apoptosis occurs mainly in the ischemic penumbra. Activation of apoptosis signaling pathways results in excessive oxygen free radical generation, DNA damage, death receptor ligation, and lysosomal protease activation. The mitochondrial apoptotic pathway is one of the predominant pathways of apoptosis (Watts et al., 2013). Studies have found that a variety of regulatory gene products involved in the process of apoptosis, including the cysteine protease (Caspases) family, Bcl-2 family, and p53 (Elmore, 2007). NF-κB and inflammasomes play an important role in the inflammatory cascade (Fann et al., 2017). Recent studies have demonstrated that in response to cerebral ischemia, NLRP3 inflammasomes promote inflammatory responses in neurons and glial cells, resulting in tissue damage (Gao et al., 2017). Increased NLRP3 is also associated with the apoptosis of neurons and glial cells. Studies have shown that activation of the NLRP3 inflammasome in brain cells can regulate the activation of caspase-1, which converts pro-IL-1β into a mature form of IL-1β (Tong et al., 2015). Given these complex mechanisms, the efficacy of a single target drug in the treatment of stroke may not be satisfactory, and, thus, it is necessary to identify a pleiotropic drug.

Anthocyanins are natural flavonoids formed by the combination of anthocyanidin and glycosides, which are widely found in the cytoplasm of flowers, fruits, stems, leaves, and root organs of plants. Studies have confirmed the biological benefits of dietary anthocyanins, as they exhibit anticancer activity, anti-inflammatory activity, neuroprotective activity, anti-obesity activity, anti-diabetic activity, and properties involved in the prevention of cardiovascular disease (Li et al., 2017). Anthocyanins can reduce neuronal damage induced by focal cerebral ischemia by blocking the JNK and p53 signaling pathways (Shin et al., 2006). Mechanically, it has been shown that anthocyanins inhibit the NF-κB signaling pathway, mitogen-activated protein kinases, and Cyclooxygenases (COXs; Li et al., 2017). Despite these findings, anthocyanins exhibit a wide range of biological activities, and their mechanisms may be diverse and largely unexplored.

In this study, we investigated the protective effects of anthocyanin in rats with MCAO/R injury, and found some clues on the underlying mechanism of protection.

## Materials and Methods

### Ethics Statement

All procedures described in this study were performed in accordance with the guidelines of Nanjing University of Chinese Medicine Animal Care and Use Committee.

### Reagents

Chromatographic grade methanol and acetonitrile were purchased from Merck (Darmstadt, Germany). Formic acid, ethyl acetate and other chemicals were purchased from commercial sources and of analytical grade. Rabbit monoclonal to Bad, rabbit monoclonal to Bcl-xl, rabbit monoclonal to Bax, rabbit polyclonal to Bcl-2, rabbit polyclonal to p-NF-κB p50, rabbit polyclonal to p-NF-κB p65, and rabbit monoclonal to p-IκB were purchased from Abcam (Cambridge, United Kingdom). Antibodies for mouse monoclonal to NLRP3 were acquired from Adipogen International (San Diego, CA, United States). Antibody for glyceraldehyde-3-phosphate dehydrogenase (GAPDH) were acquired from Sigma-Aldrich (St. Louis, MO, United States). Evans blue (EB) was purchased from Sigma Chemical Co., Ltd. (St. Louis, MO, United States). All other reagents were obtained from Sigma-Aldrich (St. Louis, MO, United States).

### Anthocyanin Preparation

Anthocyanin, namely pentunidin-3-*O*-rutinoside (*p*-coumaroyl)-5-*O*-glucoside (purity 98.3%) was extracted and purified from the dried fruits of *Lycium ruthenicum* Murr.(LRM, sampled from Delingha, Qinhai, China) according to the method proposed by Wang et al. (2014) with slight modification.

Briefly, anthocyanin was extracted twice from the dried fruits of LRM using 1% formic acid (40°C) at a ratio of 1:30 for 1 h each time. The crude extract of anthocyanins was subjected to liquid–liquid extraction, four times by water-saturated ethyl acetate at a ratio of 1:3 for 12 h each time, and was purified using a column (1 cm × 15 cm) of Amberlite XAD-7HP macroporous adsorption resin (Sigma-Aldrich, St. Louis, MO, United States). Then, two fractions of anthocyanins were isolated from the Amberlite XAD-7HP eluate using a column (1 cm × 100 cm) of Sephadex LH-20 (GE Healthcare, Chicago, IL, United States). Subsequently, the monomeric anthocyanin of pentunidin-3-*O*-rutinoside (*p*-coumaroyl)-5-*O*-glucoside was separated from the second fraction of the Sephadex LH-20 eluate by an Alltech 1500 semi-preparative HPLC (Chicago, IL, United States) and was identified using an AB SCIEX API 4000 ESI-MS/MS (Framingham, MA, United States).

### Animals

Adult male Sprague–Dawley rats weighing 250–300 g were provided by Model Animal Research Center of Nanjing University. All animals were housed five per cage under controlled temperature (22 ± 2°C) and with 12 h light/dark cycle (lights on at 8:00 a.m.). The animals had free access to food and water. Rats were injected intraperitoneally with different doses of anthocyanin (50, 100, and 200 mg/kg) for 7 days before the induction of middle cerebral artery occlusion.

### MCAO/R Model

The common carotid artery was isolated following rat anesthesia (10% chloral hydrate, 350 mg/kg, intraperitoneally), and the branches of the right external carotid artery were carefully separated. A 4-0 nylon monofilament suture with a silicon coated tip was advanced to the internal carotid artery to occlude the source of the MCA. In the sham group, this same procedure was performed but no sutures were inserted. After 2 h of cerebral ischemia, the suture was withdrawn for reperfusion for 24 h.

### Evaluation of Neurological Deficits

Each rat was evaluated for neurological deficits according to Longa’s method (Longa et al., 1989), which included the following neurological scores and symptoms: 0, no obvious neurological symptoms; 1, the rat’s front paw cannot be fully extended; 2, the rat circles to one side while walking; 3, the rat leans to one side while walking; and 4, rats cannot walk or lose consciousness.

### Evaluation of Cognitive Function

Each rat was evaluated for cognitive function by novel object recognition test, 24 h before the test, the mice were habituated to the arenas (50 cm × 50 cm plastic container) for 5 min without objects. The day after the mice re-entered the arenas from the same starting point of the arena (facing the bottom left corner) and granted 10 min to familiarize themselves with the two identical objects. Exactly 1 h after the familiarization period, the rats are exposed to two different object: one is same with the training phase and another is a novel object. The time that the rats spend to explore the old and novel objects are recorded, calculated the object interaction ratio according to the formula: object interaction ratio = Novel object exploration time/Old object exploration time.

### Evaluation of Infarct Volume

After 2 h of MCAO and 24 h of reperfusion, the brain was quickly removed. Infarct volume was then measured by TTC staining, which revealed the severity of cerebral ischemia. The data was expressed as a percentage of the total hemisphere (Lin et al., 1993).

### Evaluation of Cerebral Edema

Brain water content was measured using a dry-wet method. Brain tissue was removed after 24 h of reperfusion, and was immediately weighed (a) It was then baked in a 120°C oven for 24 h and weighed again (b) Brain water content was measured according to the formula: brain water content = (a–b)/a × 100%.

### Evaluation of BBB Permeability

The rats in each group were injected with 2% EB (2 ml/kg) in the tail vein 2 h before sacrifice. The brain tissue was then removed and weighed, and then placed into dimethylformamide (1 ml/100 mg brain tissue) and incubated at 60°C. for 24 h. After centrifugation at 1000 r/min for 5 min, the supernatant was taken and the absorbance at a wavelength of 620 nm was measured with a spectrophotometer.

### Gelatin Zymography

Protein concentrations were determined after samples collection. The samples, mixed with loading buffer, were loaded into the wells of a precast gel and subjected to electrophoresis. Then the gel was washed in washing solution followed by shaking in incubation solution for 48 h (37.5°C). After staining with Coomassie Brilliant Blue, the gel was bleached by different concentrations of methanol-acetic acid.

### Serum ELISA Detection

Samples of 0.5 ml of blood were obtained from the femoral vein at 24 h after reperfusion. The serum was separated and collected by centrifugation. Then the serum samples were then assessed for inflammatory mediator (TNF-α, IL-1β, and IL-6) levels using an ELISA kit, according to the manufacturer’s instructions.

### Western Blot

Tissues were placed in a pre-cooled glass grinder, a pre-cooled lysate containing PMSF and phosphatase inhibitors was added, and they were thoroughly ground and lysed. An appropriate amount of 5X protein loading buffer was added to the protein sample. The protein (50 μg) was separated by SDS-PAGE and transferred to PVDF membranes. The membranes were blocked with 5% bovine serum albumin at room temperature for 1 h and then incubated with a specific primary antibody overnight at 4°C. The following primary antibodies used: Bad (1:2000), Bax (1:1000), Bcl-2 (1:1000), Bcl-xl (1:1000), p-NF-κB p50 (1:1000), p-NF-κB p65 (1:2000), p-IκB (1:5000), and NLRP3 (1:500). The corresponding secondary antibody was incubated for 1 h at room temperature and the blot was visualized using the enhanced chemiluminescence (ECL) method. Software Quantity One -4.6.5 software (Bio-Rad Laboratories, Irvine, CA, United States) was used to analyze the data.

### Real Time-PCR

Trizol reagent was added to the sample, which was homogenized, and the RNA was extracted. cDNA was then synthesized and SYBR Green I dye was used for real-time quantitative PCR. The sense and antisense primers used for the analysis of rat TNF-α, IL-1β, IL-6, and GAPDH expression were as follows. TNF-a: 5′-CCC CTTTATCGTCTACTCCTC-3′ and 5′-GCTGGTAGTTTAGCTCCGTTT-3′ (553 bp). IL-1b: 5′-TCATTGTGGCTGTGGAGAAG-3′ and 5′-CTATGTCCCGACCATTGCTG-3′ (579 bp). IL-6: 5′-GGATACCACCCACAACAGAC-3′ and 5′-TTGCCGAGTAGACCTCATAG-3′ (520 bp). GAPDH: 5′-CCATCACTGCCACTCAGAAGA-3′ and 5′-CATGAGGTCCACCACCCTGT-3′ (446 bp).

### Histomorphological Analysis

After the tissue was formed into a block of wax, it was cut into 4 μm slices, dewaxed, and hydrated. It was stained with hematoxylin for 4 min and eosin for 90 s. It was dehydrated with an alcohol gradient from low to high concentrations, made transparent with xylene, and then observed under the microscope (Li et al., 2002).

### TUNEL Staining

The terminal deoxy transferase reaction mixture was incubated with the sections for 1 h according to the manufacturer’s instruction. All of the counted nuclei were stained with hematoxylin.

### Statistical Analysis

Statistical analysis was carried out using ANOVA. Results are expressed as mean ± SD. A *p* value < 0.05 was considered statistically significant. All analyses were performed using GraphPad Prism Version 5.01 software (GraphPad Software Inc., San Diego, CA, United States).

## Results

### Anthocyanin Decreased MCAO/R-Induced Brain Infarct Volume and Improved Neurologic Functional Outcomes

**FIGURE 1:**
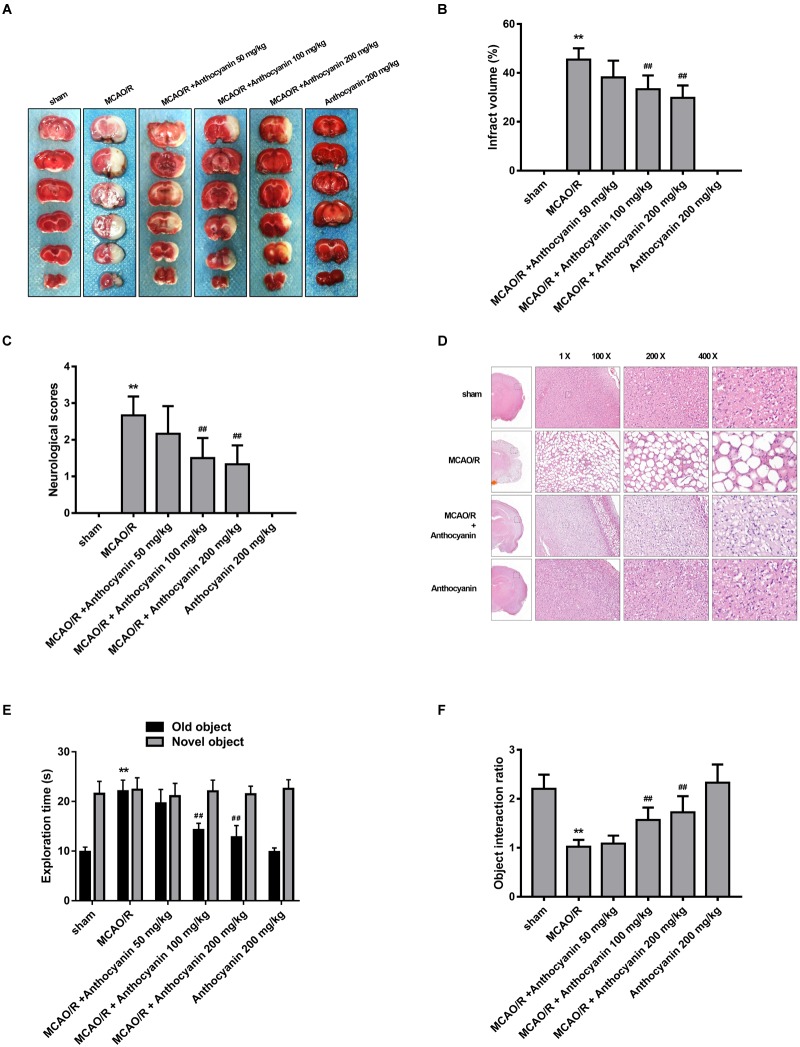
Anthocyanin reduces infarct volume and improves neurological deficits. **(A)** TTC staining of the brains of rats. **(B)** Quantitative analysis of infarct volume of rats. *F*(5,30) = 111.2. **(C)** Quantitative analysis of neurological deficits scores in rats. *F*(5,30) = 31.05. **(D)** Hematoxylin and eosin (H&E) staining of brain sections of rats. **(E)** The exploration time of novel object recognition test in rats. *F*(5,30) = 42.84 (old object). *F*(5,30) = 0.413 (novel object). **(F)** The object interaction ratio of novel object recognition test in rats. *F*(5,30) = 24.28, *n* = 6, ^∗∗^*p* < 0.01 vs. sham; ^##^*p* < 0.01 vs. middle cerebral artery occlusion/reperfusion (MCAO/R).

To evaluate the protective effect of anthocyanin, different concentrations of anthocyanin (50, 100, and 200 mg/kg) were injected intraperitoneally into rats before the MCAO/R surgery. The infarct area of the brain was examined by 2,3,5-Triphenyltetrazolium chloride staining. MCAO/R produced massive infarction, however, in the MCAO/R rats that received anthocyanin injections, the infarct volume was found to be significantly reduced in a dose-dependent manner (Figures 1A,B). Additionally, behavioral assessment showed that anthocyanin significantly decreased the neurological severity scores and attenuated cognitive function decline measured by novel object recognition test of MCAO/R rats in a dose-dependent manner (Figures 1C,E,F). Therefore, we used anthocyanin 200 mg/kg for subsequent experiments—the dose that resulted in the greatest effect. In addition, as shown in Figure 1D, neuronal loss and the presence of numerous vacuolated spaces were observed in MCAO/R rats, which could be ameliorated by anthocyanin (200 mg/kg).

### Anthocyanin Inhibited MCAO/R-Induced BBB Destruction by Inhibiting MMP-9

**FIGURE 2:**
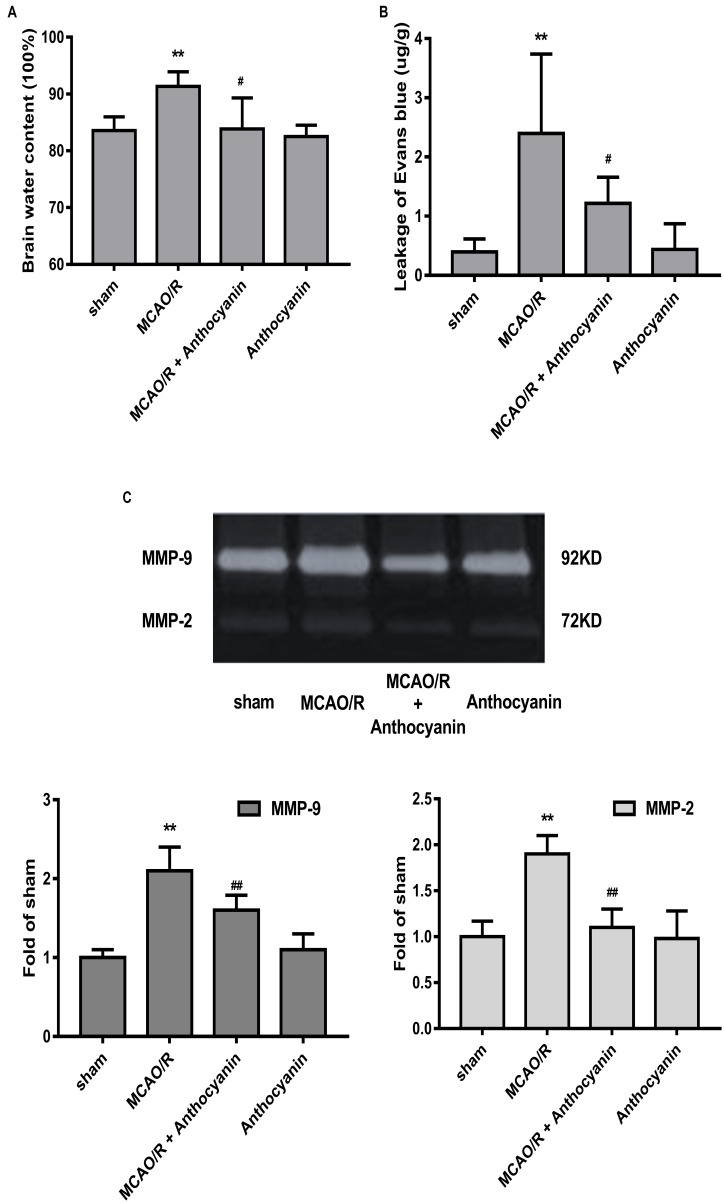
Anthocyanin reduced MCAO/R-induced blood-brain barrier (BBB) destruction by inhibiting MMP-9. **(A)** Quantitation of brain water contents in rats following MCAO/R. *F*(3,20) = 8.54. **(B)** Quantitative analysis of EB extravasation by spectrophotometry. *F*(3,20) = 9.473. **(C)** Representative band with gelatin zymography and quantitative analysis of the expression levels of MMP-9/2 in rats following MCAO/R. *F*(3,20) = 49.27 (MMP-9), *F*(3,20) = 24.39 (MMP-2), *n* = 6, ^∗∗^*p* < 0.01 vs. sham; ^#^*p* < 0.05 and ^##^*p* < 0.01 vs. MCAO/R.

Blood-brain barrier is the core structure of the NVU, which is damaged in MCAO/R. Therefore, we next examined brain edema and leakage, which is believed to reflect the permeability of the BBB. The results show that cerebral edema in the anthocyanin-treated rats was significantly alleviated compared with MCAO/R rats (Figure 2A). Similarly, anthocyanin significantly reduced the leakage of EB content (Figure 2B).

Many studies have confirmed that MMP-9 causes BBB damage in cerebral ischemia (Kurzepa et al., 2014). Therefore, we examined the effect of anthocyanin on MMP-9 levels. Gelatin zymography results showed that MCAO/R induced a significant increase in MMP-9, which could be reduced by anthocyanin treatment (200 mg/kg) (Figure 2C).

### Mitochondria Are Involved in the Anti-apoptotic Effect of Anthocyanin

**FIGURE 3:**
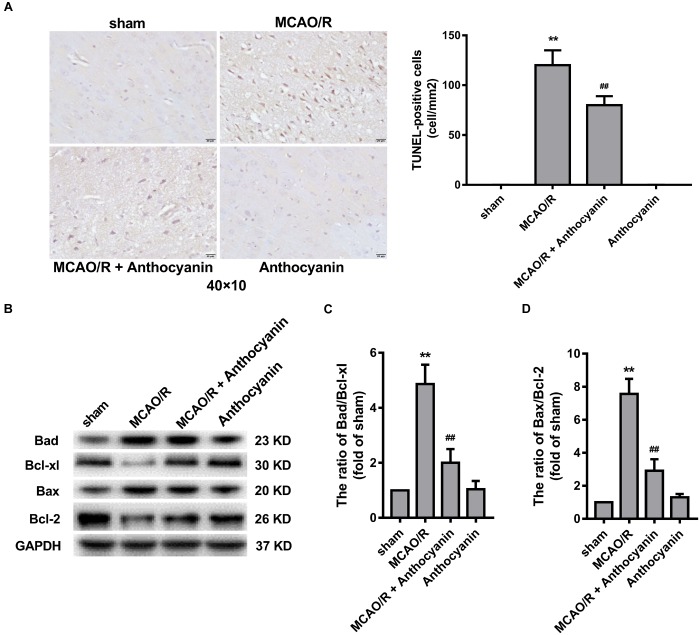
Bcl-2 family proteins are involved in the anti-apoptosis effect of anthocyanin. **(A)** Representative photomicrographs show TUNEL staining for apoptotic cells in rat brains. Effects on the severity of cerebral apoptosis are shown in an average quantitative analysis of the number of TUNEL-positive cells. *F*(3,20) = 295. **(B)** Western blot analysis of the expression of Bad, Bcl-xl, Bax, and Bcl-2 in rats. **(C,D)** The expression ratio of Bad/Bcl-xl and Bax/Bcl-2 was quantified by Quantity One 4.6.5 software and is represented as a histogram. *F*(3,20) = 136.4 (Bad/Bcl-xl), *F*(3,20) = 136.7 (Bax/Bcl-2), *n* = 6, ^∗∗^*p* < 0.01 vs. sham; ^##^*p* < 0.01 vs. MCAO/R.

Many studies have reported that the Bcl-2 family is an important apoptosis regulator in the mitochondria (Gross, 2016). A large number of TUNEL-positive cells was observed in the MCAO/R rats, and anthocyanidin (200 mg/kg) significantly reduced the number of TUNEL-positive cells, as shown in Figure 3A. In addition, anthocyanin significantly increased the Bcl-xl/Bad and Bcl-2/Bax ratios (Figures 3B–D), suggesting that it had attenuated the apoptosis signaling pathway by modulating mitochondrial function.

### Anthocyanin Inhibits MCAO/R-Induced Inflammatory Molecule Expression

**FIGURE 4:**
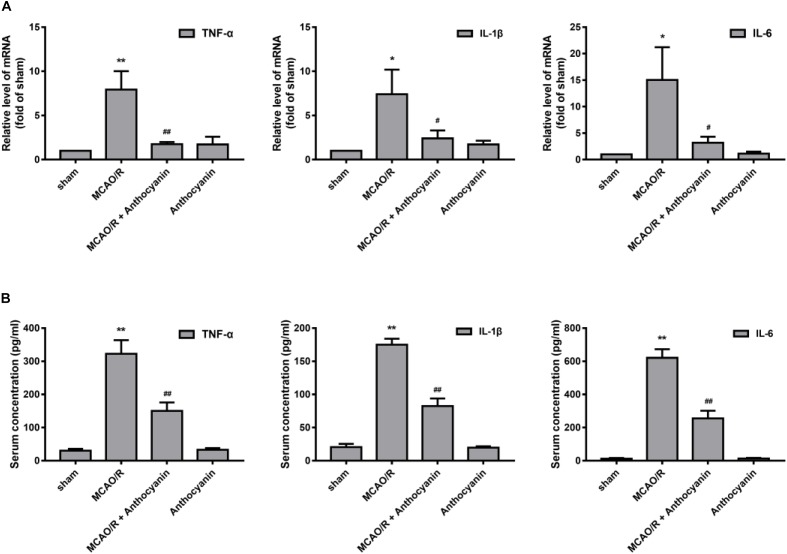
Anthocyanin inhibited the expression of inflammation-related molecules in the brain and serum of MCAO/R rats. **(A)** The effects of anthocyanin on mRNA expression levels of proinflammatory markers: TNF-α, IL-1β, and IL-6. GAPDH was used as a loading control. *F*(3,20) = 60.87 (TNF-α), *F*(3,20) = 28.8 (IL-1β), *F*(3,20) = 33.79 (IL-6). **(B)** Serum concentrations of TNF-α, IL-1β, and IL-6 in MCAO/R rats are determined. *F*(3,20) = 235.6 (TNF-α), *F*(3,20) = 649.3 (IL-1β), *F*(3,20) = 509.6 (IL-6), *n* = 6, ^∗^*p* < 0.05 and ^∗∗^*p* < 0.01 vs. sham; ^#^*p* < 0.05 and ^##^*p* < 0.01 vs. MCAO/R.

Next, we examined the effect of anthocyanin on the inflammatory response. The results showed that compared with the sham rats, MCAO/R significantly up-regulated the expression of TNF-α, IL-1β, and IL-6 in the cerebral cortex, and anthocyanin significantly inhibited the expression of TNF-α, IL-1β, and IL-6 (Figure 4A). We then used ELISA to measure serum TNF-α, IL-1β, and IL-6 concentrations. The results showed that anthocyanin significantly reduced MCAO/R-induced elevated levels of TNF-α, IL-1β, and IL-6, which is consistent with the mRNA results (Figure 4B). These findings suggest that anthocyanin could inhibit the expression of inflammation-related molecules in rat brains, induced by MCAO/R.

### NF-κB and the NLRP3 Inflammasome Pathway Are Involved in the Anti-inflammatory Effect of Anthocyanin

**FIGURE 5:**
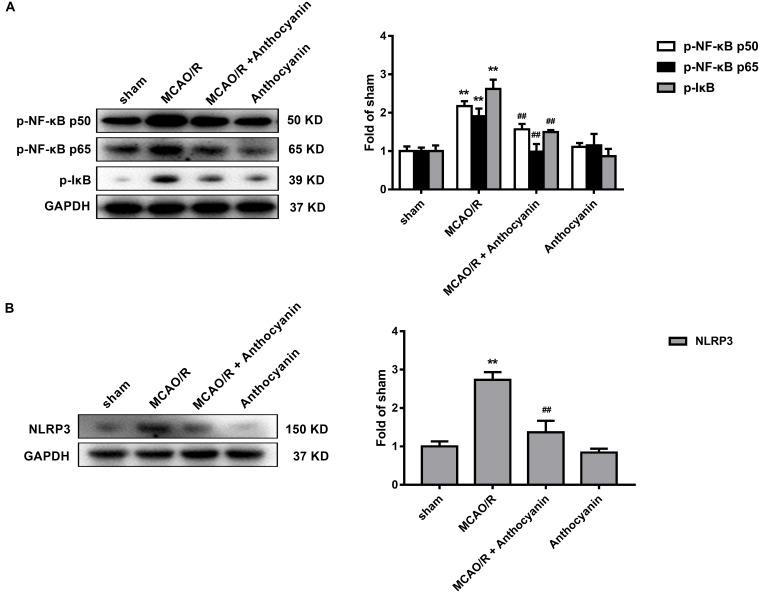
NF-κB and the NLRP3 inflammasome pathway are involved in the anti-inflammatory effect of anthocyanin. **(A)** Western blot analysis of the expression of p-NF-κB p50, p-NF-κB p65, and p-IkB in rats. *F*(3,20) = 71.53 (p-NF-κB p50), *F*(3,20) = 74.84 (p-NF-κB p65), *F*(3,20) = 184.2 (p-IkB). **(B)** Western blot analysis of the expression of NLRP3 in rats. *F*(3,20) = 175.6, *n* = 6, ^∗∗^*p* < 0.01 vs. sham; ^##^*p* < 0.01 vs. MCAO/R.

**FIGURE 6:**
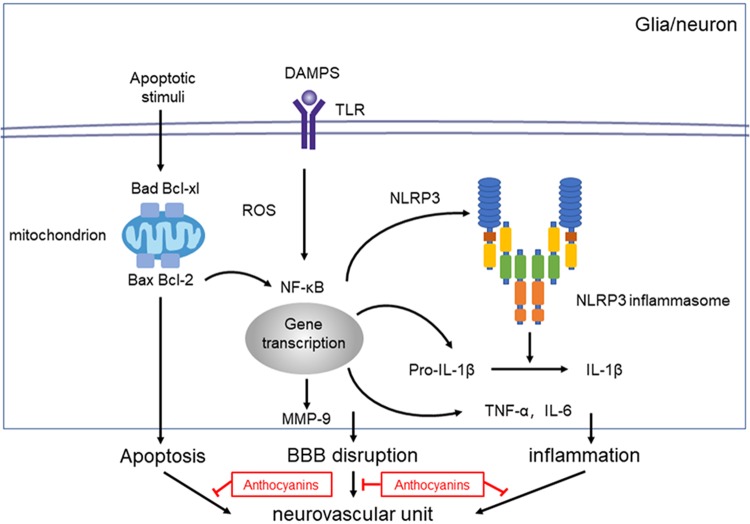
Proposed mechanisms of anthocyanin’s effect on NVU dysfunction after ischemic stroke. Injury to the neurovascular unit results in either death or injury to endothelial cells, neurons, microglia etc. Following hypoxia and ischemia, reactive oxygen species (ROS) are generated and NF-κB are activated, which activates the NLRP3 inflammasome pathway, and triggers the expression of IL-1β. Anthocyanin ameliorates the ischemia-hypoxia-induced BBB disruption by down regulating the expression of MMP-9. Anthocyanin down-regulate the expression level of Bax/Bcl-2 and Bad/Bcl-xl, which ultimately inhibits the extrinsic and intrinsic apoptotic signaling pathways to block apoptosis.

Evidence shows that NF-κB and the NLRP3 inflammasome pathway play an important role in the inflammatory signaling pathways (Dolunay et al., 2017). To identify whether NF-κB and NLRP3 are also involved in the anthocyanin-induced anti-inflammatory effect following MCAO/R, we performed western blot analysis. As shown in Figure 5, anthocyanin reduced the expression of p-NF-κB p50, p-NF-κB p65, p-IκB, and NLRP3 following MCAO/R. Based on the obtained results, the mechanisms of action and the underlying signaling pathways of anthocyanin are proposed in Figure 6.

## Discussion

Our study demonstrated that the administration of anthocyanin significantly reduced infarct volume, neurological scores, cognitive function decline, cerebral edema and the concentration of EB in MCAO/R rats by inhibiting MMP-9, Bcl-2 family and inflammation-related molecules including TNF-α, IL-1β, and IL-6. In addition, NF-κB and the NLRP3 inflammasome pathway may play a role in the anti-inflammatory effect of anthocyanin. Taken together, these results may enhance our understanding of the NVU protection properties of anthocyanin against ischemic stroke through multiple pathways and provide an alternative option for the future prevention and treatment of stroke.

Stroke is one of the most common causes of death worldwide and is a major cause of acquired disability in adults, with highly complex pathological process (Soler and Ruiz, 2010). In recent years, the NVU has become a new target for stroke treatment (Hu et al., 2017). NVU is composed of neurons, the BBB, glial cells (astrocytes, microglia), vascular cells (endothelial cells), and the ECM. The BBB is the core structure of the NVU, and matrix metalloproteinases (MMPs) have been widely considered to play a key role in the disruption of the BBB following stroke (Liu et al., 2012). In the early phase of stroke, MMPs cause multiple NVU dysfunctions, such as neuronal death and BBB leakage (Rosell and Lo, 2008).

Previous studies have shown that MMP-9 is abnormally expressed in brains suffering from cerebral ischemic injury and that it promotes brain damage and BBB breakdown (Rosenberg and Yang, 2007). In humans, patients with ischemic stroke show higher levels of MMP-9 in the blood, and more importantly, MMP-9 levels are associated with a poorer prognosis (Dejonckheere et al., 2011). Specifically, inhibitors or gene suppression of MMP-9, significantly reduced infarct size and bleeding complications (Hu et al., 2009). Our results indicate that anthocyanin may protect BBB permeability through the inhibition MMP-9. Consistent with our results, studies that administered MMP inhibitors prior to stroke showned similar benefits (Asahi et al., 2000).

Blood-brain barrier dysfunction caused by apoptosis and inflammation, is a key factor that causes NVU injury, which promotes the development of ischemic brain injury (Lakhan et al., 2009). Apoptosis contributes to a significant proportion of neuronal death that occurs following brain ischemia (Radak et al., 2017). The Bcl-2 protein family is composed of pro-apoptotic (Bax, Bad) and anti-apoptotic (Bcl-2, Bcl-xl) members, which are major regulators of the mitochondrial apoptotic pathway (Gross, 2016), and are involved in the occurrence and development of stroke (Qi et al., 2015). In this study, anthocyanin alleviated apoptosis injury resulting from MCAO/R. Additionally, anthocyanin increased levels of Bcl-2 and Bcl-xl in MCAO/R rats, and, it decreased levels of Bax and Bad. This finding suggests that anthocyanin can protect the NVU by inhibiting apoptosis, and therefore, may be a potential candidate for the treatment of ischemia-reperfusion injury.

After stroke, cerebral ischemia injury induces a robust inflammatory response, causing great damage to the BBB. Damage-associated molecular patterns (DAMPs) are the main signals that trigger inflammatory reactions. Most DAMPs, including HMGB-1 or heat shock proteins, are released from dying and dead cells (Chen and Nunez, 2010), and are sensed by pattern recognition receptors, including Toll-like receptors (TLRs) and scavenger receptors. Once the DAMP-receptor signaling is activated, inflammatory mediators such as cytokines (IL-1β, TNF-α, and IL-6), chemokines and reactive oxygen species (ROS) are released from the NVU component cells to exacerbate cell death and lead to BBB breakdown (Macrez et al., 2011). The NF-κB transcriptional activation pathway is considered to be a master regulator of inflammation and is indeed critical to the regulation of apoptosis, and play a key role in stroke (Harari and Liao, 2010). Decreasing NF-κB expression could reduce stroke size, edema, and neurological deficits in the rat permanent MCAO model (Wang et al., 2010). Furthermore, NF-κB can regulate the expression of MMP-9 (Wang et al., 2009). Inhibitors of MMP-9 can significantly reduce cerebral edema and BBB disruption in stroke (Wang et al., 2009). Our results showed that anthocyanin significantly decreased the expression of p-NF-κB p65, p-NF-κB p50, and MMP-9, suggesting NF-κB pathway is involved in the NVU protection effect of anthocyanin.

One study found that the expression of NLRP3 was increased in the brain tissue of stroke patients (Fann et al., 2013). The activation of TLR-4 activates NF-κB during cerebral ischemia, and NF-κB further promotes the production of NLRP3 and IL-1β— thus, enhancing inflammation (Minutoli et al., 2016). The NLRP3 inflammasome has become a critical mediator of post-ischemic inflammation, which eventually leads to cell death (Yang et al., 2014). NLRP3 was expressed in the neurons, microglia, and endothelial cells of ischemic brains, all of which primarily make up the NVU (Famakin, 2014). In primary cortical neurons, ischemic conditions increased NLRP3 expression and IL-1β levels. Similarly, levels of NLRP3 and IL-1β were elevated in the brains of I/R mice (Fann et al., 2013). In addition, studies have shown that in a mouse MCAO model, NLRP3^−/−^ mice exhibited reduced infarct volumes, decreased edema formation, and preserved BBB permeability (Fernandez-Cadenas et al., 2012; Lambertsen et al., 2012). In this study, our results showed that anthocyanin could inhibit MCAO/R-induced up regulation of NLRP3 in the ischemic brains of rats, indicating that anthocyanin protects the NVU against inflammation, at least in part, through a mechanism mediated by NLRP3 signaling.

Anthocyanins, which are flavonoids formed by anthocyanidins and sugars through glycosidic bonds, are natural antioxidants with a wide range of biomedical functions. Anthocyanins are thought to benefit cardiovascular, inflammatory, and degenerative diseases (Zafra-Stone et al., 2007). Anthocyanins are recognized as ROS scavengers, and endogenous ROS are mainly produced by the mitochondrial respiratory chain and the NADPH oxidase system (Dikalov, 2011). Mitochondria and NADPH oxidase have previously been implicated in BBB dysfunction and its loss of integrity (Doll et al., 2015; Schiavone et al., 2017). Studies have shown that anthocyanins have pharmacological effects on mitochondria cardioprotection through the following mechanisms: reduction of cytosolic cytochrome c preventing apoptosis and sustainment of electron transfer between NADH dehydrogenase and cytochrome c supporting oxidative phosphorylation in ischemia-damaged mitochondria (Liobikas et al., 2016). However, further research is required to investigate the role of different free radical sources in the inhibition of cerebral ischemia by anthocyanins.

Although anthocyanins have considerable beneficial effects, we still need to pay attention to the safety of anthocyanins. Anthocyanins do not appear to be readily absorbed or metabolized. A weight-of-evidence analysis indicates that anthocyanins are not genotoxic (Haveland-Smith, 1981; Ferguson et al., 1985). The acute oral toxicity is low; for instance, the oral LD_50_ for a combination of the glycosides of cyanidin, delphinidin, petundin, and malvidin was more than 25 g/kg for mice and more than 20 g/kg for rats (Hallagan et al., 1995). However, no study gives the lethal dose of anthocyanins in the case of intraperitoneal injection. In our study, the anthocyanin were intraperitoneally injected into rats by the concentrations of 50, 100, and 200 mg/kg. It may be concluded by the oral studies that intraperitoneal injection of anthocyanins may be safe in a relatively lower dose, and some other scholars have reported the benefits of intraperitoneal injection of anthocyanins in other doses (Rossi et al., 2003; Matsumoto et al., 2006; Khan et al., 2016). Despite all of these, we still need to focus some sights to the intraperitoneal injection security issues of anthocyanins, and further research is required to investigate the safe dosage of intraperitoneal injection of anthocyanins.

In addition, the anthocyanin attenuated progression of cerebral ischemic damage in rats was, associated with the suppression of the JNK and p53 signaling pathway (Shin et al., 2006). Our study found that anthocyanin could also inhibit MMP-9 and NLRP3 levels to facilitate NVU protection. Therefore, anthocyanin may potentially be a safe and effective treatment for improving stroke through multiple-effects.

Taken together, the NVU is important in stroke pathophysiology, and the investigation of multiple-pathways that protect of the NVU may lead to novel approaches for this devastating disease. Our study found that anthocyanin could inhibit apoptosis and inflammation to protect the NVU in rats impaired by MCAO/R. Additionally, NF-κB, MMP-9, and the NLRP3 inflammasome pathway might be involved anthocyanin’s protective effects in the NVU. Therefore, anthocyanin could protect the NVU through multiple pathways, and might be a potential candidate for the prevention and treatment of stroke.

## Data Availability Statement

Data and materials will be available from the authors upon reasonable request and with the permission of ZP after submission of the thesis.

## Author Contributions

ZP carried out the experiments and assisted with data analysis. MC and GD helped with the manuscript preparation and the *in vivo* experiments. TY helped with the design of the study. YL conducted the RT-PCR experiment and assisted with the manuscript preparation. TJ performed the Western Blot experiment and assisted with the data analysis. YP helped conduct the experiments and contributed to the writing of the manuscript.

## Conflict of Interest Statement

The authors declare that the research was conducted in the absence of any commercial or financial relationships that could be construed as a potential conflict of interest.

## References

[B1] Asahi M. Asahi K. Wang X. Lo E. H. (2000). Reduction of tissue plasminogen activator-induced hemorrhage and brain injury by free radical spin trapping after embolic focal cerebral ischemia in rats. *J. Cereb. Blood Flow Metab.* 20 452–457. 10.1097/00004647-200003000-00002 10724108

[B2] Chen G. Y. Nunez G. (2010). Sterile inflammation: sensing and reacting to damage. *Nat. Rev. Immunol.* 10 826–837. 10.1038/nri2873 21088683 PMC3114424

[B3] Dejonckheere E. Vandenbroucke R. E. Libert C. (2011). Matrix metalloproteinases as drug targets in ischemia/reperfusion injury. *Drug Discov. Today* 16 762–778. 10.1016/j.drudis.2011.06.009 21745586

[B4] Dikalov S. (2011). Cross talk between mitochondria and NADPH oxidases. *Free Radic. Biol. Med.* 51 1289–1301. 10.1016/j.freeradbiomed.2011.06.033 21777669 PMC3163726

[B5] Doll D. N. Hu H. Sun J. Lewis S. E. Simpkins J. W. Ren X. (2015). Mitochondrial crisis in cerebrovascular endothelial cells opens the blood-brain barrier. *Stroke* 46 1681–1689. 10.1161/STROKEAHA.115.009099 25922503 PMC4418219

[B6] Dolunay A. Senol S. P. Temiz-Resitoglu M. Guden D. S. Sari A. N. Sahan-Firat S. (2017). Inhibition of NLRP3 inflammasome prevents lps-induced inflammatory hyperalgesia in mice: contribution of NF-kappaB, caspase-1/11, ASC, NOX, and NOS Isoforms. *Inflammation* 40 366–386. 10.1007/s10753-016-0483-3 27924425

[B7] Elmore S. (2007). Apoptosis: a review of programmed cell death. *Toxicol. Pathol.* 35 495–516. 10.1080/01926230701320337 17562483 PMC2117903

[B8] Famakin B. M. (2014). The immune response to acute focal cerebral ischemia and associated post-stroke immunodepression: a focused review. *Aging Dis.* 5 307–326. 10.14336/AD.2014.0500307 25276490 PMC4173797

[B9] Fann D. Y. Lee S. Y. Manzanero S. Tang S. C. Gelderblom M. Chunduri P. (2013). Intravenous immunoglobulin suppresses NLRP1 and NLRP3 inflammasome-mediated neuronal death in ischemic stroke. *Cell Death Dis.* 4:e790. 10.1038/cddis.2013.326 24008734 PMC3789184

[B10] Fann D. Y. Lim Y. A. Cheng Y. L. Lok K. Z. Chunduri P. Baik S. H. (2017). Evidence that NF-κB and MAPK signaling promotes NLRP inflammasome activation in neurons following ischemic stroke. *Mol. Neurobiol.* 55 1082–1096. 10.1007/s12035-017-0394-9 28092085

[B11] Ferguson L. R. van Zijl P. Holloway W. D. Jones W. T. (1985). Condensed tannins induce micronuclei in cultured V79 Chinese hamster cells. *Mutat. Res.* 158 89–95. 10.1016/0165-1218(85)90102-8 3900722

[B12] Fernandez-Cadenas I. Del Rio-Espinola A. Giralt D. Domingues-Montanari S. Quiroga A. Mendioroz M. (2012). IL1B and VWF variants are associated with fibrinolytic early recanalization in patients with ischemic stroke. *Stroke* 43 2659–2665. 10.1161/STROKEAHA.112.657007 22858724

[B13] Gao L. Dong Q. Song Z. Shen F. Shi J. Li Y. (2017). NLRP3 inflammasome: a promising target in ischemic stroke. *Inflamm. Res.* 66 17–24. 10.1007/s00011-016-0981-7 27576327

[B14] Gross A. (2016). BCL-2 family proteins as regulators of mitochondria metabolism. *Biochim. Biophys. Acta* 1857 1243–1246. 10.1016/j.bbabio.2016.01.017 26827940

[B15] Hallagan J. B. Allen D. C. Borzelleca J. F. (1995). The safety and regulatory status of food, drug and cosmetics colour additives exempt from certification. *Food Chem. Toxicol.* 33 515–528. 10.1016/0278-6915(95)00010-Y 7797179

[B16] Harari O. A. Liao J. K. (2010). NF-kappaB and innate immunity in ischemic stroke. *Ann. N. Y. Acad. Sci.* 1207 32–40. 10.1111/j.1749-6632.2010.05735.x 20955423 PMC3807097

[B17] Haveland-Smith R. B. (1981). Evaluation of the genotoxicity of some natural food colours using bacterial assays. *Mutat. Res.* 91 285–290. 10.1016/0165-7992(81)90002-6 7022199

[B18] Hu Q. Chen C. Yan J. Yang X. Shi X. Zhao J. (2009). Therapeutic application of gene silencing MMP-9 in a middle cerebral artery occlusion-induced focal ischemia rat model. *Exp. Neurol.* 216 35–46. 10.1016/j.expneurol.2008.11.007 19073180

[B19] Hu X. De Silva T. M. Chen J. Faraci F. M. (2017). Cerebral vascular disease and neurovascular injury in ischemic stroke. *Circ. Res.* 120 449–471. 10.1161/CIRCRESAHA.116.308427 28154097 PMC5313039

[B20] Khan M. S. Ali T. Kim M. W. Jo M. H. Jo M. G. Badshah H. (2016). Anthocyanins protect against LPS-induced oxidative stress-mediated neuroinflammation and neurodegeneration in the adult mouse cortex. *Neurochem. Int.* 100 1–10. 10.1016/j.neuint.2016.08.005 27522965

[B21] Kurzepa J. Kurzepa J. Golab P. Czerska S. Bielewicz J. (2014). The significance of matrix metalloproteinase (MMP)-2 and MMP-9 in the ischemic stroke. *Int. J. Neurosci.* 124 707–716. 10.3109/00207454.2013.872102 24304146

[B22] Lake E. M. Bazzigaluppi P. Mester J. Thomason L. A. Janik R. Brown M. (2017). Neurovascular unit remodelling in the subacute stage of stroke recovery. *Neuroimage* 146 869–882. 10.1016/j.neuroimage.2016.09.016 27664828

[B23] Lakhan S. E. Kirchgessner A. Hofer M. (2009). Inflammatory mechanisms in ischemic stroke: therapeutic approaches. *J. Transl. Med.* 7:97. 10.1186/1479-5876-7-97 19919699 PMC2780998

[B24] Lambertsen K. L. Biber K. Finsen B. (2012). Inflammatory cytokines in experimental and human stroke. *J. Cereb. Blood Flow Metab.* 32 1677–1698. 10.1038/jcbfm.2012.88 22739623 PMC3434626

[B25] Li D. Wang P. Luo Y. Zhao M. Chen F. (2017). Health benefits of anthocyanins and molecular mechanisms: update from recent decade. *Crit. Rev. Food Sci. Nutr.* 57 1729–1741. 10.1080/10408398.2015.1030064 26192537

[B26] Li Y. Chen J. Chen X. G. Wang L. Gautam S. C. Xu Y. X. (2002). Human marrow stromal cell therapy for stroke in rat: neurotrophins and functional recovery. *Neurology* 59 514–523. 10.1212/WNL.59.4.514 12196642

[B27] Lin T. N. He Y. Y. Wu G. Khan M. Hsu C. Y. (1993). Effect of brain edema on infarct volume in a focal cerebral ischemia model in rats. *Stroke* 24 117–121. 10.1161/01.STR.24.1.117 8418534

[B28] Liobikas J. Skemiene K. Trumbeckaite S. Borutaite V. (2016). Anthocyanins in cardioprotection: a path through mitochondria. *Pharmacol. Res.* 113 808–815. 10.1016/j.phrs.2016.03.036 27038533

[B29] Liu J. Jin X. Liu K. J. Liu W. (2012). Matrix metalloproteinase-2-mediated occludin degradation and caveolin-1-mediated claudin-5 redistribution contribute to blood-brain barrier damage in early ischemic stroke stage. *J. Neurosci.* 32 3044–3057. 10.1523/JNEUROSCI.6409-11.2012 22378877 PMC3339570

[B30] Longa E. Z. Weinstein P. R. Carlson S. Cummins R. (1989). Reversible middle cerebral artery occlusion without craniectomy in rats. *Stroke* 20 84–91. 10.1161/01.STR.20.1.84 2643202

[B31] Macrez R. Ali C. Toutirais O. Le Mauff B. Defer G. Dirnagl U. (2011). Stroke and the immune system: from pathophysiology to new therapeutic strategies. *Lancet Neurol.* 10 471–480. 10.1016/S1474-4422(11)70066-7 21511199

[B32] Maki T. Hayakawa K. Pham L. D. Xing C. Lo E. H. Arai K. (2013). Biphasic mechanisms of neurovascular unit injury and protection in CNS diseases. *CNS Neurol Disord. Drug Targets* 12 302–315. 10.2174/1871527311312030004 23469847 PMC3845030

[B33] Matsumoto H. Nakamura Y. Iida H. Ito K. Ohguro H. (2006). Comparative assessment of distribution of blackcurrant anthocyanins in rabbit and rat ocular tissues. *Exp. Eye Res.* 83 348–356. 10.1016/j.exer.2005.12.019 16635490

[B34] Minutoli L. Puzzolo D. Rinaldi M. Irrera N. Marini H. Arcoraci V. (2016). ROS-Mediated NLRP3 Inflammasome Activation in Brain, Heart, Kidney, and Testis Ischemia/Reperfusion Injury. *Oxid. Med. Cell. Longev.* 2016:2183026. 10.1155/2016/2183026 27127546 PMC4835650

[B35] Qi Z. Dong W. Shi W. Wang R. Zhang C. Zhao Y. (2015). Bcl-2 phosphorylation triggers autophagy switch and reduces mitochondrial damage in limb remote ischemic conditioned rats after ischemic stroke. *Transl. Stroke Res.* 6 198–206. 10.1007/s12975-015-0393-y 25744447

[B36] Radak D. Katsiki N. Resanovic I. Jovanovic A. Sudar-Milovanovic E. Zafirovic S. (2017). Apoptosis and acute brain ischemia in ischemic stroke. *Curr. Vasc. Pharmacol.* 15 115–122. 10.2174/1570161115666161104095522 27823556

[B37] Rosell A. Lo E. H. (2008). Multiphasic roles for matrix metalloproteinases after stroke. *Curr. Opin. Pharmacol.* 8 82–89. 10.1016/j.coph.2007.12.001 18226583

[B38] Rosenberg G. A. (2012). Neurological diseases in relation to the blood-brain barrier. *J. Cereb. Blood Flow Metab.* 32 1139–1151. 10.1038/jcbfm.2011.197 22252235 PMC3390801

[B39] Rosenberg G. A. Yang Y. (2007). Vasogenic edema due to tight junction disruption by matrix metalloproteinases in cerebral ischemia. *Neurosurg. Focus* 22:E4 10.3171/foc.2007.22.5.5 17613235

[B40] Rossi A. Serraino I. Dugo P. Di Paola R. Mondello L. Genovese T. (2003). Protective effects of anthocyanins from blackberry in a rat model of acute lung inflammation. *Free Radic. Res.* 37 891–900. 10.1080/1071576031000112690 14567449

[B41] Schiavone S. Mhillaj E. Neri M. Morgese M. G. Tucci P. Bove M. (2017). Early loss of blood-brain barrier integrity precedes NOX2 elevation in the prefrontal cortex of an animal model of psychosis. *Mol. Neurobiol.* 54 2031–2044. 10.1007/s12035-016-9791-8 26910819 PMC5355521

[B42] Shin W. H. Park S. J. Kim E. J. (2006). Protective effect of anthocyanins in middle cerebral artery occlusion and reperfusion model of cerebral ischemia in rats. *Life Sci.* 79 130–137. 10.1016/j.lfs.2005.12.033 16442129

[B43] Soler E. P. Ruiz V. C. (2010). Epidemiology and risk factors of cerebral ischemia and ischemic heart diseases: similarities and differences. *Curr. Cardiol. Rev.* 6 138–149. 10.2174/157340310791658785 21804773 PMC2994106

[B44] Tong Y. Ding Z. H. Zhan F. X. Cai L. Yin X. Ling J. L. (2015). The NLRP3 inflammasome and stroke. *Int. J. Clin. Exp. Med.* 8 4787–4794. 26131053 PMC4483817

[B45] Turner R. J. Sharp F. R. (2016). Implications of MMP9 for blood brain barrier disruption and hemorrhagic transformation following ischemic stroke. *Front. Cell Neurosci.* 10:56. 10.3389/fncel.2016.00056 26973468 PMC4777722

[B46] Vidale S. Consoli A. Arnaboldi M. Consoli D. (2017). Postischemic inflammation in acute stroke. *J. Clin. Neurol.* 13 1–9. 10.3988/jcn.2017.13.1.1 28079313 PMC5242162

[B47] Wang E. Yin Y. Xu C. Liu J. (2014). Isolation of high-purity anthocyanin mixtures and monomers from blueberries using combined chromatographic techniques. *J. Chromatogr. A* 1327 39–48. 10.1016/j.chroma.2013.12.070 24433700

[B48] Wang G. Guo Q. Hossain M. Fazio V. Zeynalov E. Janigro D. (2009). Bone marrow-derived cells are the major source of MMP-9 contributing to blood-brain barrier dysfunction and infarct formation after ischemic stroke in mice. *Brain Res.* 1294 183–192. 10.1016/j.brainres.2009.07.070 19646426 PMC2758551

[B49] Wang L. Zhang X. Liu L. Yang R. Cui L. Li M. (2010). Atorvastatin protects rat brains against permanent focal ischemia and downregulates HMGB1, HMGB1 receptors (RAGE and TLR4), NF-kappaB expression. *Neurosci. Lett.* 471 152–156. 10.1016/j.neulet.2010.01.030 20100543

[B50] Wang Q. van Hoecke M. Tang X. N. Lee H. Zheng Z. Swanson R. A. (2009). Pyruvate protects against experimental stroke via an anti-inflammatory mechanism. *Neurobiol. Dis.* 36 223–231. 10.1016/j.nbd.2009.07.018 19635562 PMC2742567

[B51] Watts L. T. Lloyd R. Garling R. J. Duong T. (2013). Stroke neuroprotection: targeting mitochondria. *Brain Sci.* 3 540–560. 10.3390/brainsci3020540 24961414 PMC4061853

[B52] Yang F. Wang Z. Wei X. Han H. Meng X. Zhang Y. (2014). NLRP3 deficiency ameliorates neurovascular damage in experimental ischemic stroke. *J. Cereb. Blood Flow Metab.* 34 660–667. 10.1038/jcbfm.2013.242 24424382 PMC3982086

[B53] Zafra-Stone S. Yasmin T. Bagchi M. Chatterjee A. Vinson J. A. Bagchi D. (2007). Berry anthocyanins as novel antioxidants in human health and disease prevention. *Mol. Nutr. Food Res.* 51 675–683. 10.1002/mnfr.200700002 17533652

